# pH is the primary determinant of the bacterial community structure in agricultural soils impacted by polycyclic aromatic hydrocarbon pollution

**DOI:** 10.1038/srep40093

**Published:** 2017-01-04

**Authors:** Yucheng Wu, Jun Zeng, Qinghe Zhu, Zhenfa Zhang, Xiangui Lin

**Affiliations:** 1 Key Laboratory of Soil Environment and Pollution Remediation, Institute of Soil Science, Chinese Academy of Sciences, Nanjing, China; 2 Joint Open Laboratory of Soil and the Environment, Hong Kong Baptist University & Institute of Soil Science, Chinese Academy of Sciences, Nanjing, China; 3 Department of Environmental Sciences and Engineering, Gillings School of Global Public Health, University of North Carolina, Chapel Hill, North Carolina, USA

## Abstract

Acidification and pollution are two major threats to agricultural ecosystems; however, microbial community responses to co-existed soil acidification and pollution remain less explored. In this study, arable soils of broad pH (4.26–8.43) and polycyclic aromatic hydrocarbon (PAH) gradients (0.18–20.68 mg kg^−1^) were collected from vegetable farmlands. Bacterial community characteristics including abundance, diversity and composition were revealed by quantitative PCR and high-throughput sequencing. The bacterial 16S rRNA gene copies significantly correlated with soil carbon and nitrogen contents, suggesting the control of nutrients accessibility on bacterial abundance. The bacterial diversity was strongly related to soil pH, with higher diversity in neutral samples and lower in acidic samples. Soil pH was also identified by an ordination analysis as important factor shaping bacterial community composition. The relative abundances of some dominant phyla varied along the pH gradient, and the enrichment of a few phylotypes suggested their adaptation to low pH condition. In contrast, at the current pollution level, PAH showed marginal effects on soil bacterial community. Overall, these findings suggest pH was the primary determinant of bacterial community in these arable soils, indicative of a more substantial influence of acidification than PAH pollution on bacteria driven ecological processes.

Microorganisms play vital roles in soil processes such as substance turnover, biogeochemical cycling and plant growth, contributing essentially to the soil ecosystem services. Soil contains a myriad of bacteria of which the diversity and community structure are crucial to soil functional stability^1^. Considerable efforts have been invested to address how bacteria respond to the changing environment, since the terrestrial ecosystems are increasingly under the pressure of human activities. Cropland is such a hotspot of anthropogenic disturbance due to the lasting input of agricultural chemicals and pollutants over recent decades, resulting in significant changes in soil characteristics as exemplified by acidification^2^ and pollution^3^, particularly in some rapidly developed areas.

Both natural and anthropogenic processes contribute to soil acidification, and the resulting pH variation is often an important factor affecting soil bacterial diversity and community composition at different geographic scales^4^,^5^,^6^. The relation between pH and bacterial diversity could be fitted by a quadratic model, normally higher diversity in neutral soils and lower in acidic and alkaline soils^7^. The considerable shifts in soil microbial community composition along pH gradients were usually observed^6^,^8^, with enrichment of acidophilic bacteria in low pH soils^9^. Similar trends in bacterial diversity and community composition were also observed in arable soils of artificial pH gradient^10^,^11^.

Soil pollution has shown extensive effects on below-ground microbes. Among the pollutants studied, polycyclic aromatic hydrocarbons (PAHs) has been found to be widespread in terrestrial environments^12^, posing considerable risks to food safety and human health. PAH pollution could be the reason for microbial diversity loss^13^, community succession^14^ and activity change^15^,^16^. On the other hand, many bacteria have the potential of assimilating and degrading PAH, or of resistance to them, and could be selected for in even heavily contaminated environment. For example, short-term exposure to high concentration of polycyclic aromatic hydrocarbon (PAH) increased copy number of Gram-positive degrading bacteria^17^. It should be noted that effects of PAH are affected by their physicochemical characteristics and their interaction with soil matrix^18^, but so far the long-term effects of PAH on soil microbes are still less known.

Both soil acidification and pollution might co-exist in arable soils, as suggested by wide occurrence of acidification^2^ and pollution (http://www.mlr.gov.cn/xwdt/jrxw/201404/t20140417_1312998.htm) in China. The community responses to overlapped acidification and PAH pollution are therefore crucial to assess the function changes in impacted ecosystems. In our previous study, we observed co-existed pH (4.26–8.05) and PAH gradients (0.18–20.68 mg kg^−1^) within soil samples collected from a farmland adjacent to a smelting plant. As revealed by fingerprinting technique, the community of Thaumarchaeota, one of major archaeal phyla in these arable soils was tightly related to soil pH rather than to PAH, suggesting variation in archaeal nitrification in this farmland^19^. With the rapid evolvement of cultivation-independent molecular research tools, it is now possible to unravel the vast diversity of soil microbes. As such, we examined the bacterial communities in agricultural soils impacted by both acidification and PAH pollution based on the Illumina MiSeq sequencing platform. The aims of the study were therefore (1) to assess the effects of pH variation and PAH pollution on bacterial diversity and community composition, and (2) identify the sensitive phylotypes to the both environmental stresses.

## Results and Discussion

### Bacterial abundance

The abundances of bacteria in soils were estimated using qPCR targeting 16S rRNA genes. The 16S rRNA gene copies ranged from 0.18 to 1.72 × 10^10^ g^−1^, and were significantly (*p* < 0.01) related to total carbon (TC), total nitrogen (TN) and nitrate (Table 1). Both PAH and pH showed little effect on 16S rRNA gene copy number, suggesting that bacterial abundance in these arable soils was mainly controlled by nutrients supply^20^.

Bacterial aromatic ring hydroxylating dioxygenases are responsible for the initial oxidation of PAH, and the encoding gene (PAH-RHDα) copies are often used as a surrogate of degrading bacterial abundance^21^. In this study, both Gram positive (GP) and negative (GN) PAH-RHDα genes were determined with qPCR; however, smearing amplification of GN PAH-RHDα gene was obtained, possibly due to the low specificity of the primers^22^. GP PAH-RHDα gene copies in the samples varied between 1.05 and 4.90 × 10^5^ g^−1^, and were moderately correlated with PAH (Table 1). However, this does not mean the enrichment of GP degraders by PAH pollution, since they could be affected by soil characteristics or the presence of plant. It has been previously observed that abundance of PAH-RHDα gene was often changed by root exudates^23^, organic matter^24^ and pH^25^. Controlled experiments with more comprehensive and sensitive tests, for example the transcription activities analysis^26^,^27^ are therefore required to reveal the effects of PAH pollution on degrader community.

### Bacterial diversity

**Figure 1:**
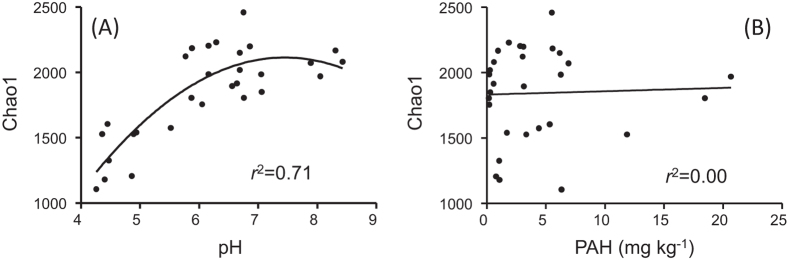
Relationship between (**A**) soil pH, (**B**) PAH and bacterial diversity as measured by the Chao 1 estimator.

The Illumina MiSeq sequencing generated 180 876 reads from 29 libraries after quality control, with an average of 6 237 reads for each sample and median length of 408-bp. The reads were clustered into OTUs based on a 97% threshold of sequence similarity. Observed species, Chao1 estimator and phylogenetic diversity were calculated, and were compared at the same level of subsampling (2 000 reads). All measures of bacterial diversity were tightly related to pH (*r* ≥ 0.7, *p* < 0.01, Table 1). The relationship between pH and bacterial richness, as measured by Chao 1 estimator, could be fitted by a quadratic model, with the highest richness observed at near neutral pH (6.0–7.0, see Fig. 1A), suggesting pH is one of the crucial determinants of bacterial diversity in this farmland. In addition, the bacterial diversity measures were moderately correlated with ammonium and total potassium (*p* < 0.01, Table 1).

Soil acidification could be caused by both natural and anthropogenic processes, but in vegetable cultivation intensive use of nitrogen fertilizers could be the primary reason for the soil pH decrease^2^. The background pH of the dominant soil type (Argosols) in this area was normally neutral, and the wide variation in soil pH observed in this study was very likely attributed to the application of mineral N fertilizer during vegetable cultivation. This is implicated by the relation between NH_4_^+^-N and pH (*r* = −0.570, *p* < 0.01), and partly addresses the link between ammonium and bacterial diversity (Table 1).

Despite a two orders of magnitude variation in soil concentration (0.18–20.68 mg kg^−1^), PAH showed negligible influence on bacterial diversity (Fig. 1B) and community composition (see below). This was not rare for naturally occurred soil pollution^28^, although PAH may inhibit the growth of pure cultured bacteria at extremely low concentration^29^. The stress of toxic chemicals on soil microbes is affected by many factors, including pollutants’ physicochemical properties and soil interaction. For example, organic pollutants are normally hydrophobic and prone to absorb to soil particles, resulting in the decreased bioavailability of PAH^18^. As such, toxicity of PAH to microbes in soil was often lower than in pure cultured systems^29^,^30^. To data, the ecological effects of PAH on microbes in soils is not well explored and further studies are needed to reveal the interaction between soil, pollutants and microbes.

### Bacterial community structure

**Figure 2:**
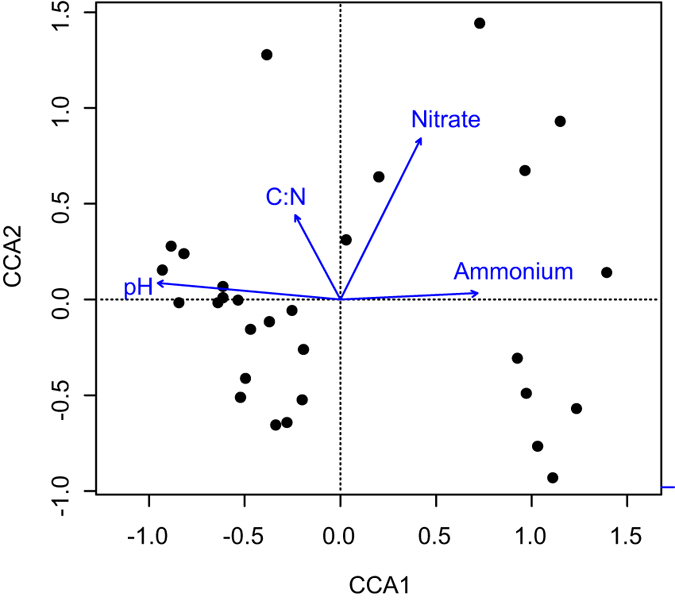
Canonical correspondence analysis (CCA) biplot of bacterial 16S rRNA genes in the arable soils showing regulating factors and samples. C/N, ratio of total carbon to nitrogen.

To identify the most important factors likely affecting the bacterial community composition, CCA was carried out with OTU table and environmental parameters. Of the 9 soil features measured, pH, C/N, ammonium and nitrate appeared to be significantly related to bacterial community structure as examined by a forward selection (*p* < 0.05), hence were included in CCA analysis. The first two axes of the CCA biplot accounted for 9.5% and 4.6% of the total community variation, respectively, with pH the most correlated factor with the first axis (Fig. 2). By contrast, the impact of PAH on bacterial community composition was minimal.

**Figure 3:**
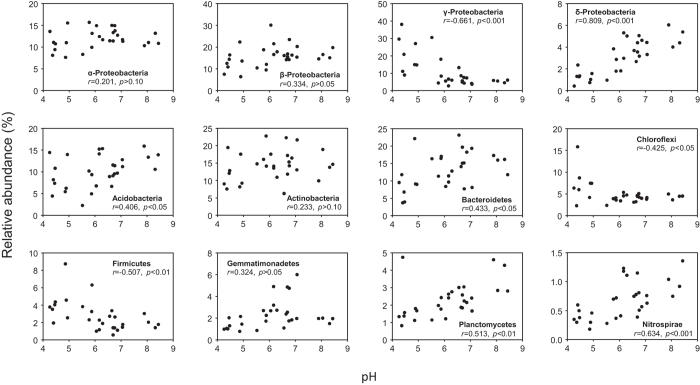
Correlations between soil pH and relative abundances of dominant bacterial phyla. Linear regressions were used to describe the relationship between the taxa’s relative abundances and pH.

**Figure 4:**
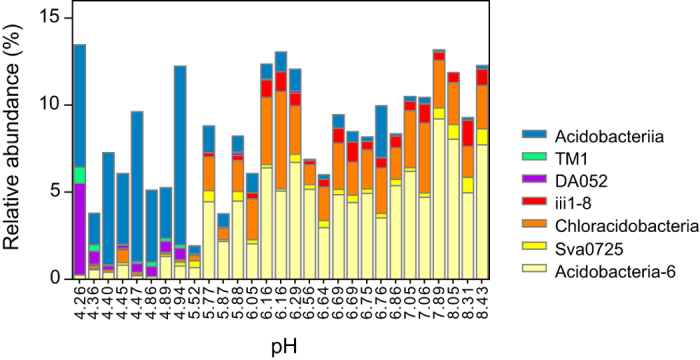
Class-level composition of Acidobacteria. The samples are arranged by their pH values.

Bacterial communities in these soils primarily consisted of Proteobacteria, Bacteroidetes, Acidobacteria and Actinobacteria, Firmicutes, Chloroflexi and Gemmatimonadetes, which accounted for >5% of the library in at least one sample. Of these dominant phyla, γ- and δ-Proteobacteria, as well as Nitrospirae which was less abundant, were closely related to pH (*p* < 0.001, Fig. 3), while PAH pollution showed little effect. Different from many previous studies, only marginal correlation between pH and the phylum Acidobacteria was observed (Fig. 3). At the level of class, however, variation in relative abundance of Acidobacterial clades was obvious along the pH gradient (Fig. 4). For example, subgroup 6 (Gp6), iii1–8 and Chloracidobacteria of Acidobacteria were enriched in slightly acidic to near neutral samples, while Acidobacteriia, TM1 and DA052 group were exclusively dominant in samples of pH < 5.0. Gp-6 of Acidobacteria dominated in many agricultural soils and was often positively correlated with soil pH^9^,^31^. By contrast, Acidobacteriia was often enriched in low-pH environment^9^, implicating an acidophilic chemoorganotrophic life style as has been described for culture *Acidobacterium capsulatum*^32^.

**Figure 5:**
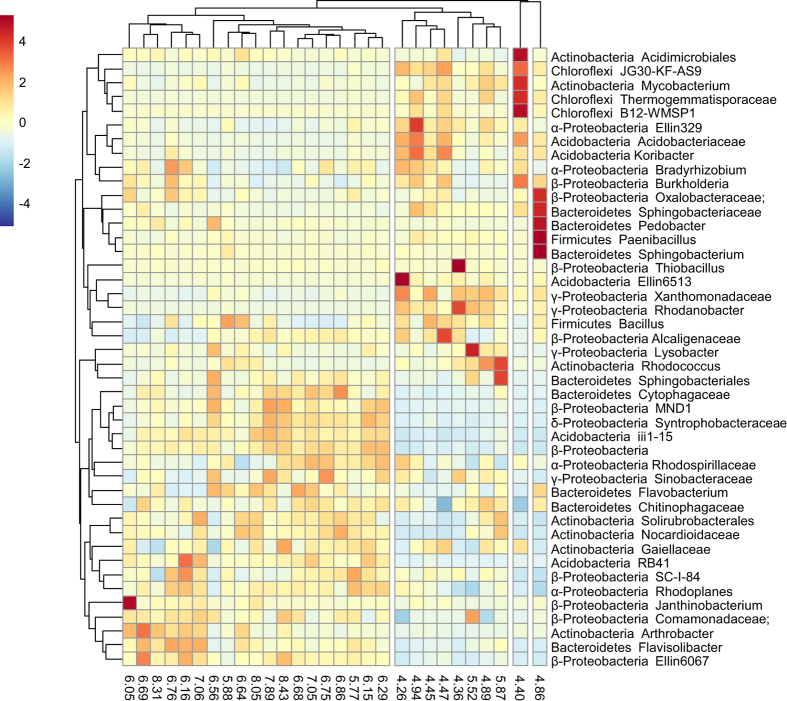
Heatmap of dominant phylotypes which were selected if their relative abundance accounted for >2% of any library. Rows representative of phylotype were centered and clustered based on Euclidean distance. The numbers at the bottom of the heatmap indicate the pH value of each sample.

The influence of pH on other dominant phylotypes could be depicted with a heatmap (Fig. 5), in which all near neutral samples (pH > 6.0) were exclusively clustered together, and the acidic soils formed separated groups. A few phylotypes of potential function could be identified as the highly responsive to pH change. For example, *Rhodanobacter* and another unknown genus of Xanthomonadaceae (γ-Proteobacteria) were highly enriched in soils of low pH, which may accounted for >30% of the library of low-pH sample (Fig. 5). *Rhodanobacter* spp. are capable of denitrification^33^, PAH degradation^34^, and inhabiting in very acidic environment^35^, suggesting they might play a role in the most acidic soils in the farmland. Among others, some phylotypes of known function were also sensitive to soil acidification. For example, there was a direct correlation between pH and *Candidatus* Nitrososphaera sequences in the MiSeq dataset (*r* = 0.634, *p* < 0.001), which is consistent to our previous study^19^ and suggests that archaea-mediated ammonia oxidation in these arable soils was strongly affected by pH. Despite the fact that the physiology of most bacterial phylotypes are unclear, these findings provide convincing evidences of essential influence of acidification on soil ecosystem functioning.

Similar correlation between PAH and individual bacterial phylotypes were found. Among others, the relative abundance of Gram positive *Rhodococcus* was directly related to PAH (*r* = 0.651, *p* < 0.001), and to copies of GP PAH-RHDα gene (*r* = 0.500, *p* < 0.05). Degradation of PAH by *Rhodococcus* sp. has been widely recognized^36^, however, whether they were actively involved in PAH transformation in the soils remains unclear. On the other hand, a few phylotypes were negatively influenced (data not shown), implicating a potential toxicity of PAH to soil bacteria, like has been revealed with pure bacterial cultures^29^. Nevertheless, further studies, in particular with controlled experiments are required to obtain the comprehensive knowledge of the ecological effects of PAH on microbes.

Taken together, this study examined bacterial abundance and structure in arable soils affected by both acidification and PAH pollution. The results reveal that pH was a primary determinant of bacterial community diversity and composition, suggesting acidification has potentially significant impacts on soil ecological processes driven by bacteria. By contrast, the influence of PAH on soil bacteria appears to be negligible within the measured pollution range, which may be in relation to the interactions among pollutants, microbes and soil matrix. Large-scale soil survey and simulated exposure to PAH will facilitate a deep insight into the ecological effect of PAH on soil microbes.

## Materials and Methods

### Soils

In total 29 soil samples were collected from three separated agricultural plots in southwest Nanjing, Jiangsu Province, China. Upon the sampling, the three plots were used for cultivation of varied vegetables. Each soils was obtained by mixing five 50-g subsamples from a 10 × 10 m grid. A 50-g subsample of each soil was stored at −20 °C for molecular analysis, and the rest was air-dried, homogenized, and stored at 4 °C for physicochemical analysis.

Major gradients were observed in the soil characteristics of the 29 samples. Among others, soil pH varied from 4.26 to 8.43 and the total amount of 15 PAHs from 0.18 to 20.68 mg kg^−1^ (Supplementary Table S1). Ten soils from the plot A (31°51′57″N, 118°35′58″E) had the highest PAH concentration, 10 soils from the adjacent plot B were relatively clean^19^, and other 9 samples from the plot C (31°53′48″N, 118°36′59″E) were moderately polluted by PAH (Supplementary Table S1). Considerable within-plot variation in pH was found, which could be attributed to intensive use of mineral nitrogen fertilizers^2^.

### Quantitative PCR (qPCR) of bacterial 16S rRNA and PAH-RHDα genes

Soil DNA was extracted using FastDNA Spin Kit for Soil following the manufacturer’s instruction. Tenfold-diluted DNA was used in the qPCR assays to avoid the inhibition of co-extracted contaminants. Bacterial 16S rRNA gene copies were determined using the primers 519f and 907r. Gram-positive (GP) PAH-ring hydroxylating dioxygenase (PAH-RHDα) genes were determined with primer sets GP-F/GP-R, respectively^21^.

QPCR was performed on a CFX96 instrument (Bio-Rad) and SYBR green-based reactions were performed in triplicate for each sample. The components of qPCR assays were described previously^37^. The amplification specificity was checked by both electrophoresis and melting curve analysis. The qPCR standards were generated using plasmid DNA from one clone containing respective gene fragments. A dilution series of the standard template across seven orders of magnitude (10^1^–10^7^ copies μl^−1^) was used per assay. The control was always run with water as the template instead of DNA extract. The qPCR amplification efficiency was 85.9% with *r*^2^-value of 0.995 for bacterial 16S rRNA gene, 71.0% with *r*^2^-value of 0.995 for GP PAH-RHDα gene.

### Illumina MiSeq sequencing of bacterial 16S rRNA gene

Bacterial community analysis was performed by sequencing bacterial 16S rRNA gene as previously described^38^. Briefly, the V4 and V5 regions of the 16S rRNA gene were amplified using the primers 519f (CAGCMGCCGCGGTAATWC) and 907r (CCGTCAATTCMTTTRAGTTT), with the former tagged a 5-nucleotide (nt) barcode. After verification by agarose gel electrophoresis, triplicated PCR products were pooled and purified. The equimolar mixture of PCR amplicons for each soil was submitted to the Center for Analysis and Test of Institute of Soil Science, CAS for sequencing using the MiSeq platform (Illumina, Inc., CA, USA).

### Sequence processing and analysis

Raw paired Illumina MiSeq data were assembled and analyzed using QIIME^39^ as described previously^40^. Reads with an average quality score of <25 were discarded. Operational taxonomic units (OTUs) were assigned using UCLUST based on a threshold of 97% sequence identity. The taxonomy of each representative of OTU was determined using the Greengenes 16S rRNA gene database (http://greengenes.lbl.gov/). In this study, 2 000 sequences per sample were randomly subsampled to calculate the Faith’s phylogenetic diversity, the observed species and the Chao 1 estimator. The correlations between microbial diversity and soil variables were calculated using SPSS 13.0. Canonical correspondence analysis (CCA) was performed with OTU table and all available environmental parameters using the vegan package of R. Heatmap was generated with centered dominant phylotypes data using the R package pheatmap, with phylotypes and samples aggregated into clusters using the complete neighbor method.

The sequences obtained in this study have been deposited in the European Molecular Biology Laboratory (EMBL) database.

## Additional Information

**How to cite this article**: Wu, Y. *et al*. pH is the primary determinant of the bacterial community structure in agricultural soils impacted by polycyclic aromatic hydrocarbon pollution. *Sci. Rep.* **7**, 40093; doi: 10.1038/srep40093 (2017).

**Publisher's note:** Springer Nature remains neutral with regard to jurisdictional claims in published maps and institutional affiliations.

## Supplementary Material

Supplementary Table

## Figures and Tables

**Table 1:** Pearson correlations between bacterial gene copies, diversity and environmental factors.

	pH	TC	TN	C/N	TP	TK	NH_4_^+^	NO_3_^−^	PAH
16S rRNA gene	0.035	0.653**	0.698**	0.266	0.289	−0.164	0.139	0.716**	0.327
GP PAH-RHDα gene	0.344	0.279	0.260	0.122	0.132	−0.201	−0.056	0.137	0.409*
Observed species	0.753**	−0.043	−0.275	0.384*	−0.431*	−0.493**	−0.652**	−0.359	0.016
Chao1	0.748**	−0.034	−0.241	0.338	−0.511**	−0.480**	−0.639**	−0.309	0.037
Phylogenetic diversity	0.697**	0.062	−0.164	0.449*	−0.410*	−0.580**	−0.654**	−0.200	0.040

TC, total carbon; TN, total nitrogen; C/N, ratio of total carbon to nitrogen; TP, total phosphorus; TK: total potassium.

* and ** indicate significance at the level of *p* < 0.05 and *p* < 0.01, res*p*ectively.
