# Supplementary material for: pH is the primary determinant of the bacterial community structure in agricultural soils impacted by polycyclic aromatic hydrocarbon pollution
Source: Sci Rep. 2017 Jan 4;7:40093. doi: 10.1038/srep40093 (PMC5209717; doi:10.1038/srep40093)
Supplement: Supplementary Table [file srep40093-s1.doc]

Supplementary Information to:
pH is the primary determinant of the bacterial community structure in agricultural soils impacted by polycyclic aromatic hydrocarbon pollution

Running title: Soil pH determines bacterial community

Yucheng Wu^1,2^, Jun Zeng^1,2^, Qinghe Zhu^1,2^, Zhenfa Zhang^3^, Xiangui Lin^1,2^*

^1^ Key Laboratory of Soil Environment and Pollution Remediation, Institute of Soil Science, Chinese Academy of Sciences, Nanjing, China
^2^ Joint Open Laboratory of Soil and the Environment, Hong Kong Baptist University & Institute of Soil Science, Chinese Academy of Sciences, Nanjing, China
^3^ Department of Environmental Sciences and Engineering, Gillings School of Global Public Health, University of North Carolina, Chapel Hill, North Carolina, USA

**Correspondence:**

Prof. Xiangui Lin

Tel: +86-25-86881589 Fax: +86-25-86881000 E-mail:xglin@issas.ac.cn

Supplementary Table

Table S1. Description of soil pH and PAH of the samples collected from three plots

|  | pH | | | |  | PAH | | | |
| --- | --- | --- | --- | --- | --- | --- | --- | --- | --- |
|  | Min. | Max. | Aver. | CV |  | Min.  (mg kg^-1^) | Max.  (mg kg^-1^) | Aver.  (mg kg^-1^) | CV |
| Plot A (n=10) | 4.26 | 8.05 | 5.77 | 0.24 |  | 3.14 | 20.68 | 8.60 | 0.73 |
| Plot B (n=10) | 4.40 | 7.06 | 5.80 | 0.18 |  | 0.18 | 1.70 | 0.64 | 0.51 |
| Plot C (n=9) | 5.77 | 8.43 | 6.92 | 0.13 |  | 0.61 | 6.28 | 3.37 | 0.64 |

CV: coefficient of variation.
